# Multi-class segmentation of neuronal structures in electron microscopy images

**DOI:** 10.1186/s12859-018-2305-0

**Published:** 2018-08-09

**Authors:** Kendrick Cetina, José M. Buenaposada, Luis Baumela

**Affiliations:** 1 Departamento de Inteligencia Artificial, Universidad Politécnica de Madrid, Campus de Montegancedo s/n, Boadilla del Monte, España, Madrid, 28660 Spain; 2 ETSII, Universidad Rey Juan Carlos, C/ Tulipán, s/n, Móstoles, 28933 Spain

**Keywords:** Image segmentation, Electron microscopy, Multi-class boosting, Neuron structures

## Abstract

**Background:**

Serial block face scanning electron microscopy (SBFEM) is becoming a popular technology in neuroscience. We have seen in the last years an increasing number of works addressing the problem of segmenting cellular structures in SBFEM images of brain tissue. The vast majority of them is designed to segment one specific structure, typically membranes, synapses and mitochondria. Our hypothesis is that the performance of these algorithms can be improved by concurrently segmenting more than one structure using image descriptions obtained at different scales.

**Results:**

We consider the simultaneous segmentation of two structures, namely, synapses with mitochondria, and mitochondra with membranes. To this end we select three image stacks encompassing different SBFEM acquisition technologies and image resolutions. We introduce both a new Boosting algorithm to perform feature scale selection and the *Jaccard Curve* as a tool compare several segmentation results. We then experimentally study the gains in performance obtained when simultaneously segmenting two structures with properly selected image descriptor scales. The results show that by doing so we achieve significant gains in segmentation accuracy when compared to the best results in the literature.

**Conclusions:**

Simultaneously segmenting several neuronal structures described at different scales provides voxel classification algorithms with highly discriminating features that significantly improve segmentation accuracy.

## Background

**Fig. 1:**
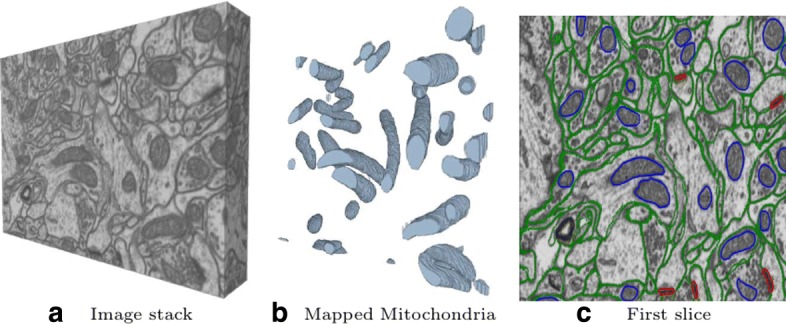
Hippocampus rat neural tissue SBFEM image. **a** image stack; **b** 3D reconstruction of mitochondria in the stack; **c** ground truth labels of mitochondria (blue) and synapses (red) in the first slice. Membrane labels (green) have been included only for illustrative purposes

Understanding the structure, connectivity and functionality of the brain is one of the challenges faced by science in the 21st century. This grand challenge is supported by the development of multiple and complementary brain imaging modalities such as structural and functional imaging [1] and light microscopy [2, 3]. At the finest level, recent advances in SFBEM also support this long term goal [4–6]. They have made it possible to automatically acquire long sequences of high resolution images of the brain at the nanometer scale. However, the automated interpretation of these images is still an open challenge, because of their inherent intricacy and its huge size (see Fig. 1a).

In this paper we consider the problem of segmenting mitochondria and synapses that along with membranes are some of the most prominent neuronal structures (see Fig. 1b and c). These structures are of interest to neuroscience. The identification and quantification of distribution of synapses provides fundamental information for the study of the brain [6]. Mitochondria, on the other hand, play a key role in the cell metabolism, physiology and pathologies [7]. The accurate segmentation and reconstruction of neuron membranes is requisite to address the neural circuit reconstruction problem [8, 9].

Given the complexity of the SBFEM images shown in Fig. 1, a fundamental step for a successful segmentation is a good feature representation. In recent years a broad range of image description features have been introduced in the literature [10]. SBFEM specialized features like Radon-like [11] and Ray features [12] along with various standard computer vision ones such as Histograms of Oriented Gradients, Local Binary Patterns and different banks of linear filters are the most usual representations [10, 13–17]. To further exploit contextual information the result of extracting these features at different scales is usually pooled in neighborhoods around the described voxels, as in [18] or in the *integral channel features* [19]. Tu and colleagues [20] use a variant of the integral channel features to segment brain 3-D magnetic resonance images. A related approach, termed *context cues*, was also used by Becker [14] and Lucchi [16] for segmenting synapses and mitochondria respectively.

Typical feature vectors have thousands of variables. For labeling these structures ensemble classification methods, in particular Boosting and random forests, are the most popular in EM segmentation approaches, since they can select the best subset of features on-the-fly, while training. Random Forest and Boosting classifiers like AdaBoost and GentleBoost have been used for segmenting synapses [13, 14, 21], membranes [22] and mitochondria [12, 16, 23].

Although there are some general software tools for segmenting neuronal structures [13, 24], the best results for synapses [14, 21, 25] and mitochondria [15, 16, 26, 27] have been achieved by algorithms specifically designed for each of them. In this paper we study whether we can improve the performance of these approaches by simultaneously segmenting more than one structure. In particular, we will concurrently segment synapses with mitochondria and mitochondria with membranes. Since these structures arise in SBFEM stacks with different sizes, we introduce a feature selection algorithm to determine the best scales to describe them. We compare our segmentations with those that target a single structure. To this end we select three image stacks and the segmentation algorithms that have reported the best performance in each of them. To make a fair evaluation we introduce a novel quality measure tool, the *Jaccard Curve*, enabling the comparison of several segmentation approaches independently of the selected operational point of the classifier.

## Methods

### Image features

**Fig. 2:**
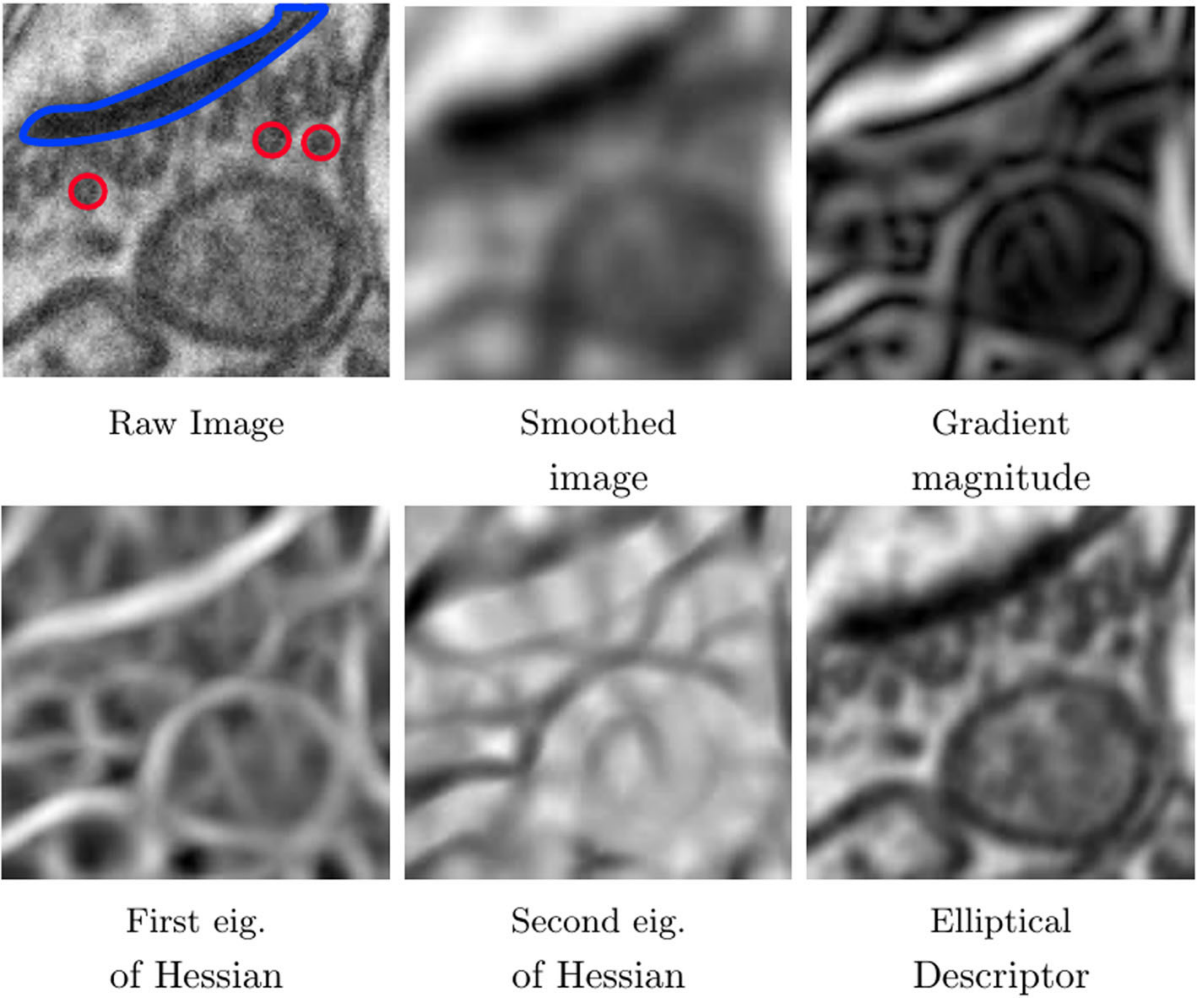
Raw image and four image description features used in our methodology. In the raw image we highlight with blue and red color a synapse and three vesicles respectively. We can appreciate the elongated shape of the synapse and the small circular shape of the vesicles, next to the synapse

We aim to use contextual information to label each voxel. To this end we use integral channel features based on extracting the sums over rectangular regions of a set of feature channels. We obtain these channels by computing a Gaussian Rotation Invariant MultiScale (GRIMS) descriptor and an elliptical descriptor at different scales. We choose GRIMS because they are an excellent descriptor for segmenting mitochondria and synapses [10, 28]. Since vesicles are a good indicator of the existence of synapses in the vicinity (see the raw image in Fig. 2), we also include an elliptical descriptor that provides contextual information related to the existence of vesicles.

GRIMS descriptors apply to each image in the stack a set of linear Gaussian filters at different scales to compute zero, first and second order derivatives, { *s*_*ijk*_:*i*+*j*+*k*≤2}, where

$$s_{ijk}=\sigma^{i+j+k}G_{\sigma}*\frac{\partial^{i+j+k}}{\partial x^{i}y^{j}z^{k}}, $$

*G*_*σ*_ is a Gaussian filter with standard deviation *σ* and ∗ is the convolution operator. We represent the result of applying these operators to the image with *s*_*ijk*_ where the summation of the subscript indices denotes the order of the derivatives. The rotation invariant feature vector at scale *σ* is given by $(s_{000}, \sqrt {s^{2}_{100} + s^{2}_{010} + s^{2}_{001}}, \lambda _{1}, \lambda _{2}, \lambda _{3})$, where the first component is the smoothed image, the second one is the magnitude of the gradient and *λ*_*i*_,*i*=1…3 are the eigenvalues of the Hessian matrix. The complete feature vector is the concatenation of all partial feature vectors at different scales *σ*_*j*_,*j*=1…*n*. Hence, the GRIMS vector has dimension 5*n*, being *n* the number of scales used to describe each voxel (see Fig. 2).

**Fig. 3:**
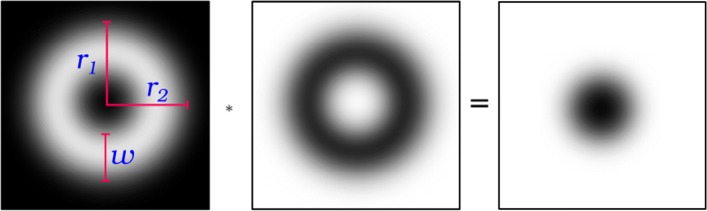
Elliptical descriptor. (left) image showing the kernel as grey values, with both radii(*r*_1_,*r*_2_) and the thickness (*w*) parameters over-imposed; (center) image of a vesicle-like structure; (right) response obtained when convolving the vesicle image with the elliptical descriptor kernel

The elliptical descriptor is the result of filtering the image with an elliptic torus-like kernel. The shape of this kernel is controlled by the radii *r*_1_, *r*_2_ and thickness *w* parameters. As shown in Fig. 3, the result of convolving this kernel (left image) with a vesicle-like structure (central image) returns low values for the inner parts of the vesicle.

### Scales selection

Properly addressing the multi-scale nature of the structures in the SBFEM images is an important issue to achieve top segmentation performance. Synapses appear in our images with various sizes and shapes. Similarly, mitochondria show up as rougly elliptical structures with very different sizes (see Figs. 1 and 9). The information provided by the features defined in the previous section depends on the size of the image structures and the scales of the kernels used for filtering. We set the parameters of the elliptical descriptor as the average radii and width of a representative set of vesicles in the stack (see Table 1). However, for a given image stack, it is not clear what is the most discriminative set of GRIMS scales. An important step in our methodology is to establish them. The standard approach would optimize the segmentation performance using cross-validation over the set of scales. However, in our problem this is computationally prohibitive. To this end we introduce a new scale selection algorithm based on a generalization of the well-known AdaBoost-based greedy feature selection scheme [29] to the multi-class case. For this purpose we adapt PIBoost [30], a recently introduced multi-class boosting algorithm with binary weak-learners (see Algorithm 1).

**Table 1:** Vesicle descriptor parameters and GRIMS scales selected for each data set

Data set	GRIMS	Vesicle parameters		
	scales	*r*_1_	*r*_2_	*w*
Hippocampus	1.2, 1.6, 5.6,6.0	5	5	2
Somatosensory cortex	1.6, 4.4, 5.6, 6.4	3	3	1
Cerebellum	1.2, 1.6, 2.0, 4.8	4	4	2

**Fig. 4:**
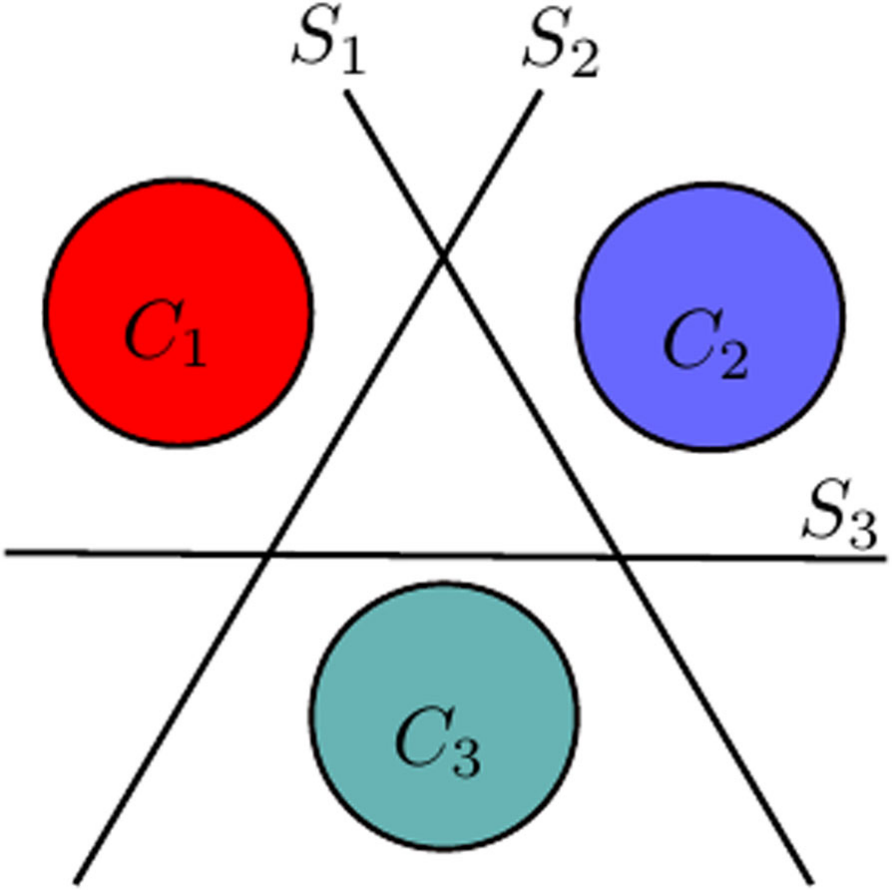
PIBoost *separator* in a three class problem. It is composed of three weak-learners (*S*_1_,*S*_2_,*S*_3_) separating each class, e.g. *C*_1_:mitochondrion, *C*_2_:synapse, *C*_3_:background, from the rest

Each PIBoost iteration learns a group of weak-learners that partially solve a multi-class classification problem. Each weak-learner separates a group of classes from the rest, learned as a binary problem in which one of the groups is treated as the positive class and the rest as negative. In this context a *separator* is a classifier formed by combining the minimal set of weak-learners that solve a multi-class problem (see Fig. 4). Each separator associates weights to training samples [30]. These weights focus the learning process on a different set of samples at each iteration thereby encouraging each weak-learner to be independent from the rest.

For feature selection we modify this scheme producing a new algorithm (see Algorithm 1). The feature selection algorithm iterates over all GRIMS scales training each separator with one scale using the weighted training samples. In our case, since we consider the simultaneous segmentation of two structures, we have two positive and one negative classes, hence, each separator has associated three weak-learners (see Fig. 4). We classify the training data with each separator (GRIMS scale) and select the one with the smallest weighted error. Finally, with the error of the selected feature we update the weights of the training data according to the PIBoost scheme [30], so that the next selected scales are independent from those selected so far. Algorithm 1 shows this process, where the actual expression of functions train *S*_*k*_(*F*_*j*_), *ε*_*j*_(*F*_*j*_,*S*_*k*,*k*=1…3_,*W*_*i*_) and *W*(*ε*_*min*_) may be found in [30], Section 4. Table 1 shows the chosen scales for each data set. They were selected among 50 equally distributed values between 1 and 50. These are the scales used in the experiments in “Results and discussion” section. So, our feature vectors have 20 components (4 selected scales, times 5 features per scale)


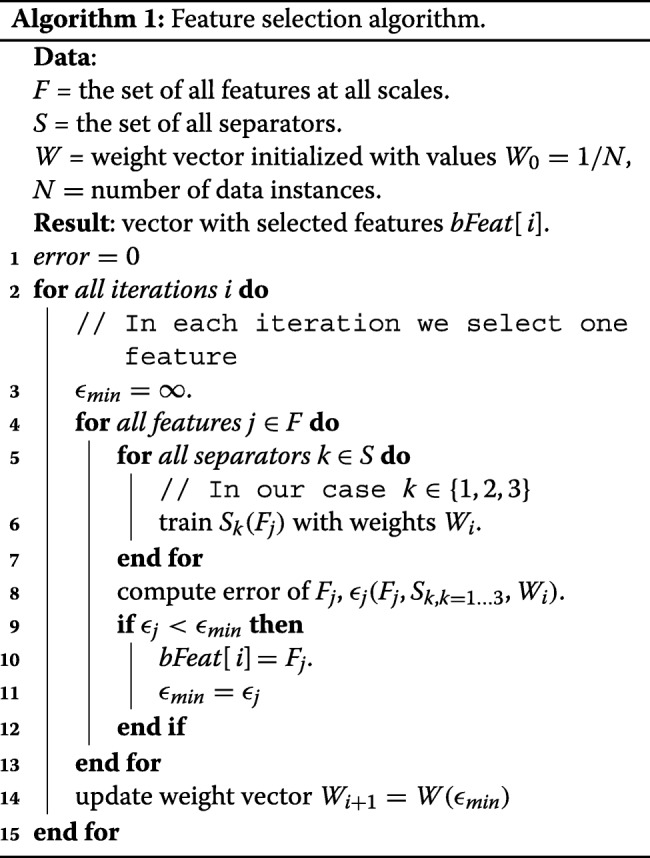


**Fig. 5:**
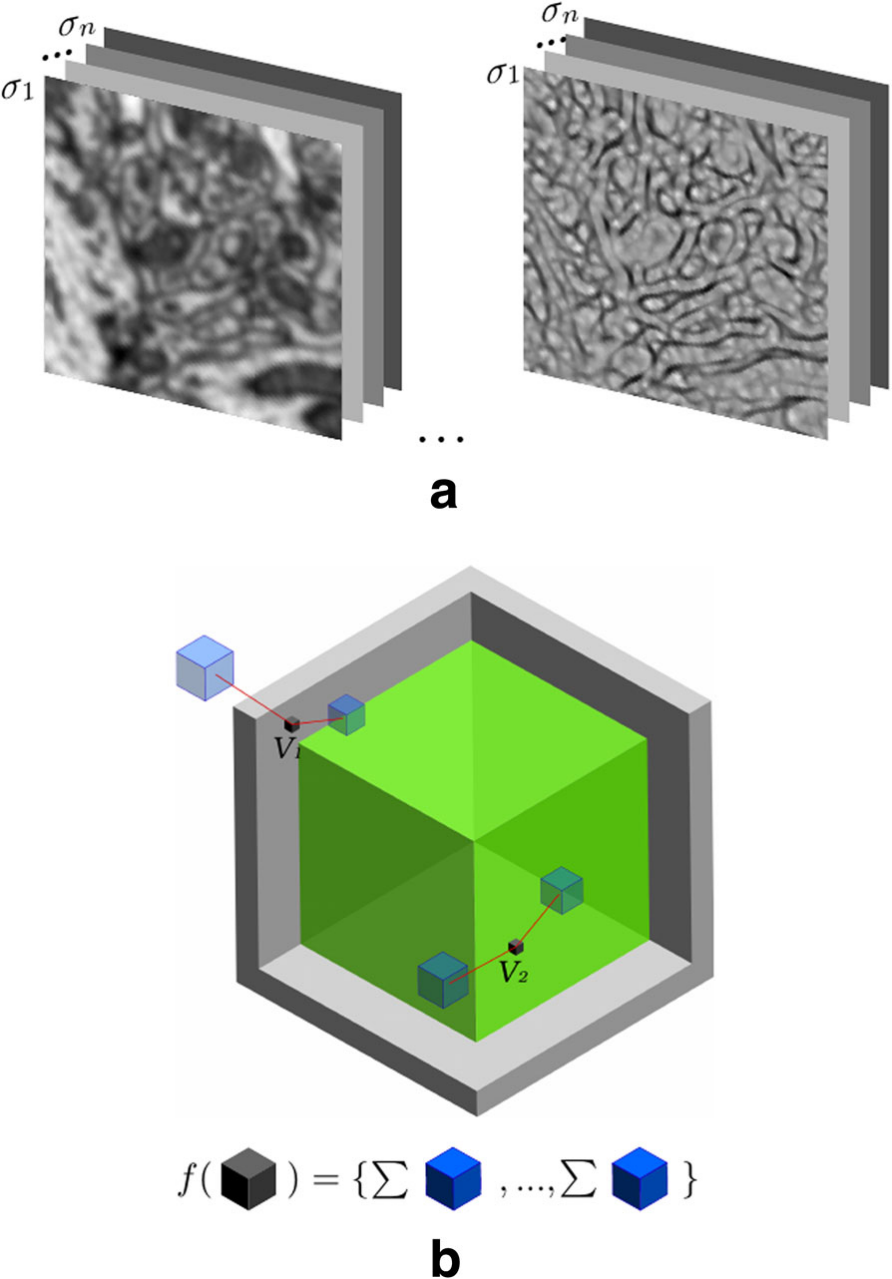
Feature extraction process. We apply a set of filters at scales *σ*_1_,…,*σ*_*n*_ to each stack slice. **a** two filters for one slice, *s*_000_ left, *λ*_3_ right; **b** a feature of voxel *V*_*i*_ is the sum of the values of one box (in blue). The feature vector of *V*_*i*_ is the concatenation of several hundreds of such features

Once selected the best features and scales, we aggregate local evidence by computing the integral channel features on them. In our approach we use cubic regions, as shown in Fig. 5b.

So, the feature extraction process is as follows. For each voxel in the stack we obtain the channels by convolving it with the GRIMS and elliptical filters in a set of selected scales, *σ*_1_,…,*σ*_*n*_ (see Fig. 5a). A feature associated to one voxel is the sum of the filter responses in a random neighboring cube in a randomly selected channel (see Fig. 5b). In our approach we extract 1200 features for each voxel in the stack.

### Multi-class boosting with integral channel features

We also adopt a Boosting scheme to label each voxel. Our classifier, Partially Informative Boosting (PIBoost) [30], is a multi-class generalization of AdaBoost with binary weak-learners.

At the *m*-th iteration and for each separator *S*, PIBoost builds a stage-wise additive model

$$\mathbf{f}_{m}(\mathbf{x})=\mathbf{f}_{m-1}(\mathbf{x})+\beta_{m}^{S}\mathbf{g}^{S}_{m}(\mathbf{x}), $$

where $\mathbf {f}_{m}(\mathbf {x})\in \mathbb {R}^{c}$ is the strong learner and $\mathbf {g}^{S}_{m}(\mathbf {x})$ the trained weak-learner at iteration *m* for separator *S*, $\beta ^{S}_{m}$ is a constant related to the accuracy of the weak-learner, and *c* is the number of classes in the problem. Each component in the vector **f**(**x**) represents to what extent **x** belongs to each class. **f**(**x**) satisfies the *sum-to-zero* condition, **f**(**x**)^⊤^**1**=0, that guarantees that each vector takes one and only one value from the set of labels [30]. Finally, sample **x** is assigned to the class *α*_*i*_ associated to the maximum component of **f**(**x**)

$$\mathbf{x}\in \alpha_{i} \Leftrightarrow i=\arg\max_{j}\mathbf{f}(\mathbf{x})[j], $$

where **f**(**x**)[*j*] denotes the *j*-th component of vector **f**(**x**).

We also use sub-sampling to optimize the classifier performance [31]. To this end, we train each weak-learner with a fraction of all training data. Sub-sampling reduces training time helps to generalize the classification. For the PIBoost experiments in “Results and discussion” section we train each weak-learner with 10% of the data randomly sampled according to their weights. This gives priority to instances that are hard to classify.

### Label regularization

The output of this classification process is noisy. We filter this noise by optimizing the energy in a Markov Random Field (MRF) with pairwise terms using the graph cut algorithm [32]. Since the standard graph cut approach is only valid for binary problems, we solve our three class regularization problem in two ways. First by setting up two one positive class-against the rest problems. Second using the *αβ-swap* multi-class extension to graph cuts [33].

We define the weights of edges in the graph as follows. Let us denote with *α*_*y*_,*y*∈{*mitochondrion*, *synapse*,*membrane*,*background*} each of the class labels for a voxel. The unary term of voxel **x** for class *α*_*j*_, *u*(**x**,*α*_*j*_) is given by the minus log of its posterior probability, *u*(**x**,*α*_*j*_)=− log*P*(*α*_*j*_∣**f**(**x**)). Using the multinomial logistic expression we get

1 $$ {\begin{aligned} -\log P(\alpha_{j} \mid \mathbf{f}(\mathbf{x})) &= -\log\frac{e^{\mathbf{f}(\mathbf{x})[j]}}{\sum_{i} e^{\mathbf{f}(\mathbf{x})[i]}}\\ &= \log \sum_{i} e^{\mathbf{f}(\mathbf{x})[i]} - \mathbf{f}(\mathbf{x})[j]. \end{aligned}}  $$

Since $\log \sum _{i} e^{\mathbf {f}(\mathbf {x})[i]}\approx \max \{\mathbf {f}(\mathbf {x})\}$, then, the unary term weights are given by

2 $$ u(\mathbf{x},\alpha_{j})= \max\{\mathbf{f}(\mathbf{x})\}-\mathbf{f}(\mathbf{x})[j].   $$

For the pair-wise terms we train a new classifier that learns the probability that a voxel belongs to a border. Here a border is a thin strip around the edge of mitochondria and synapses. This is done by setting up a PIBoost-based classifier with only two classes *α*_*y*_,*y*∈{*border*,*no*_*border*}. The weight of the edge connecting neighboring voxels **x** and **y**, *p*(**x**,**y**), is given by

3 $$ p(\mathbf{x},\mathbf{y})=-\log P(\alpha_{border}|\mathbf{f}(\mathbf{x})) -\log P(\alpha_{border}|\mathbf{f}(\mathbf{y})).   $$

## Results and discussion

Here we describe the experiments performed to evaluate the image segmentation method described in the previous section.

### Quality measure

**Fig. 6:**
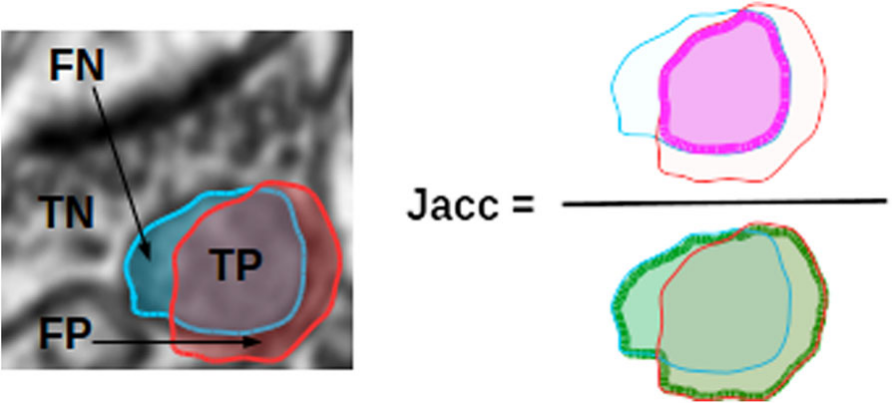
Jaccard similarity coefficient. (left) in blue the ground truth segmentation of a mitochondrion, in red the result obtained with an automated segmentation algorithm; TP is the intersection of red and blue regions, i.e. the correctly segmented piece of mitochondrion; FN is the only blue area, i.e. the part of mitochondrion segmented as background; FP is the only red area, i.e. the background segmented as mitocondrion; TN is the rest of the image, i.e. the correctly segmented background; (right) representation of the Jaccard coefficient as the relation between the purple and green areas

We use as measure of quality of a segmentation the *Jaccard* similarity coefficient between the ground truth and the result provided by the algorithm evaluated. It is a widely used image segmentation quality index both in the computer vision and bio-medical literature [15, 27]. It is defined as the area of the intersection divided by the area of the union of segmentations (see Fig. 6). In terms of classification results it can be expressed as

$$ JAC=\frac{TP}{TP+FP+FN}, $$

where TP stands for true positive, FP false positive and FN false negative. It represents a binary non-symmetric measure of coincidence of two segmentations. It takes values between 0 (no coincidence) and 1 (total coincidence). In our results Jaccard indices are computed from each positive class (mitochondria, synapses, membranes) versus the rest. Although this is the most usual way to show results, other works compute the average Jaccard index of positive and negative classes [27].

In binary classification problems a threshold value controls how posterior probabilities are converted into class labels. To compare the performance of two such classifiers independently of the threshold the Machine Learning community has long agreed on the use of Precision Recall (PR) or Receiver Operator Characteristic (ROC) curves instead of accuracy results [34]. Similarly, in image segmentation, simply comparing a Jaccard index may be inaccurate, since, for example, the same classification algorithm with a different classification threshold would exhibit different Jaccards.

**Fig. 7:**
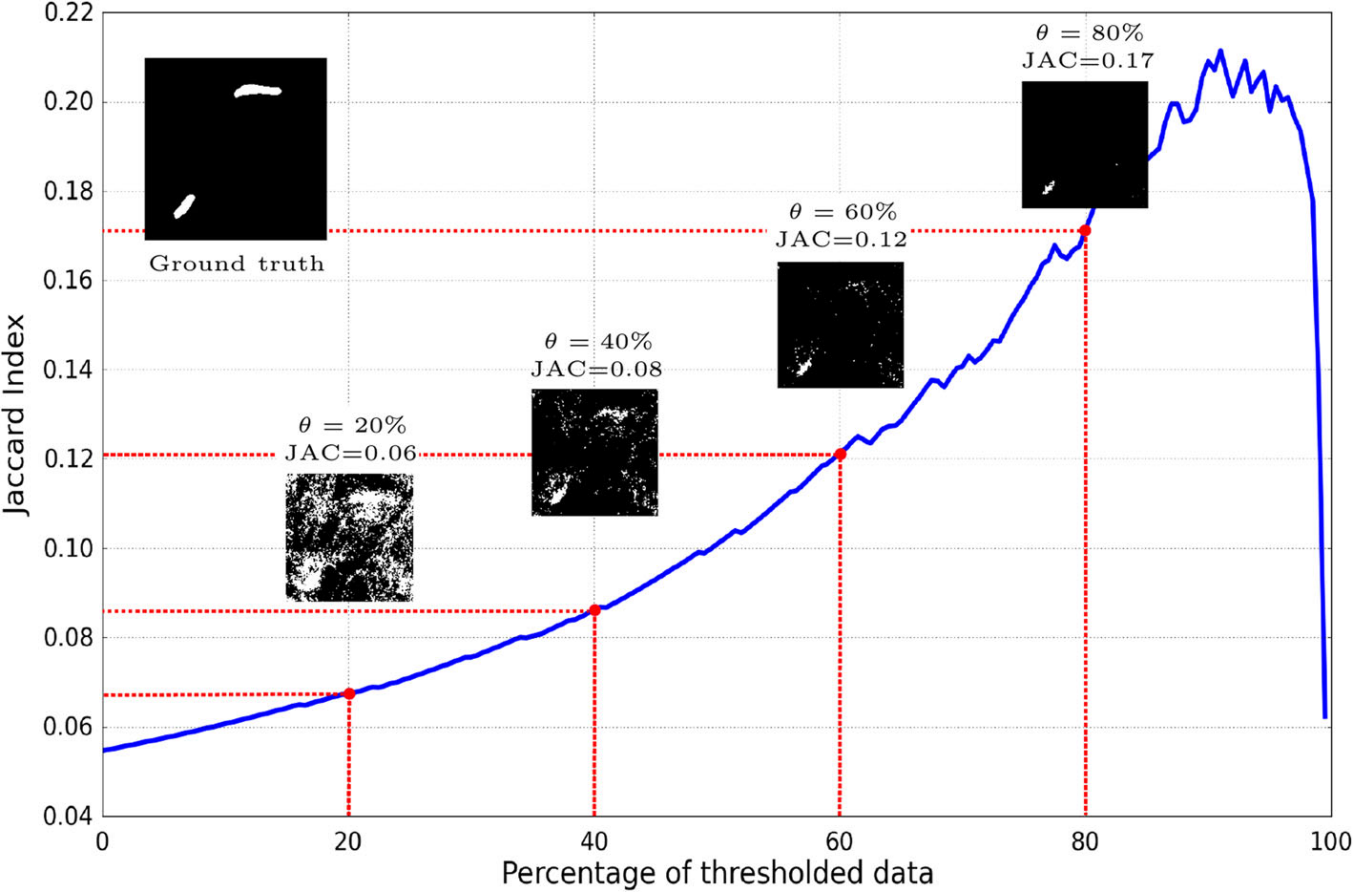
Example of a Jaccard curve obtained with the results of the segmentation of two synapses. We selected a hard-to-segment patch to see the segmentation improvement with different thresholds. In the vertical axis we represent the Jaccard (JAC), whereas in the horizontal the percentage of pixels segmented as background. We can see how the higher the threshold (*θ*) the less elements are segmented as synapse

Here we introduce the *Jaccard Curve* (JCC) as a means of comparing the performance of two segmentation algorithms independently of their classification threshold. In the horizontal axis of the JCC we represent the proportion of pixels below the positive class score threshold, i.e. the percentage of pixels in the image labeled as background. In the vertical one we plot the Jaccard of the segmentation obtained when labeling in the positive class all voxels with a score higher or equal to the threshold (see Fig. 7). We plot the JCC by sorting all voxels according to their score and evaluating the Jaccard of the segmentations at different thresholds. The higher the JCC curve, the better the segmentation.

The evaluation of membrane segmentation using the Jaccard index has been criticized because it is commonly believed that small deviations in the detected membrane locations are acceptable, which however will cause large errors in the estimated Jaccard index. Alternative more robust metrics such as the *Rand F-score*(${\mathcal {F}}_{r}$) and *Information Theoretic F-score*(${\mathcal {F}}_{it}$) have been recently proposed [35]. For membrane segmentation we will also use these these metrics.

### Experiments

In our experiments we have used three serial section electron microscopy data sets comprising different labels, SBFEM acquisition technologies and levels of anisotropy (see Table 2). The first two stacks, Hippocampus and Somatosensory cortex, were acquired with FIB-SEM microscopes. The have synapses and mitochondria labels manually annotated by expert neuroanatomists^1^. The Hippocampus stack has perfectly isotropic voxels with very high resolution. The rat Somatosensory Cortex one has a coarser resolution with slightly an-isotropic voxels. Finally, the Cerebellum stack^2^, was acquired with a SBF-SEM microscope. It has mitochondria and membranes labels with the largest *anisotropy factor* [28].

For our analysis we select the algorithms reporting the best results for each of the selected stacks. We compare our algorithm with the AdaBoost-based approach of Lucci et al. for segmenting mitochondria [36], and Becker et al. for segmenting synapses [14]. We also compare our algorithm with the Bayesian approach of Marquez et al. [28], that segments both structures. To this end, we use the code provided by the authors. For the experiments with AdaBoost we trained the algorithm with 1200 decision stumps based on *context cues* [14, 16]. For the Bayesian approach we trained a classifier with Gaussian class-conditional distributions and GRIMS features as described in [28]. Finally, for PIBoost we conducted 50 iterations training 150 decision tree weak-learners. The input to this classifier are pooled features in cubes of size 5×5×5 voxels computed on the channels extracted from GRIMS and elliptical descriptors on the set of selected scales, as described in “Methods” section. In all our experiments we used the first block of consecutive slices of each stack for training, and the rest for testing (see Table 2). Since the number of voxels from the background class is much larger than that of the two other positive classes, when training PIBoost we randomly discard half of the background voxels.

**Fig. 8:**
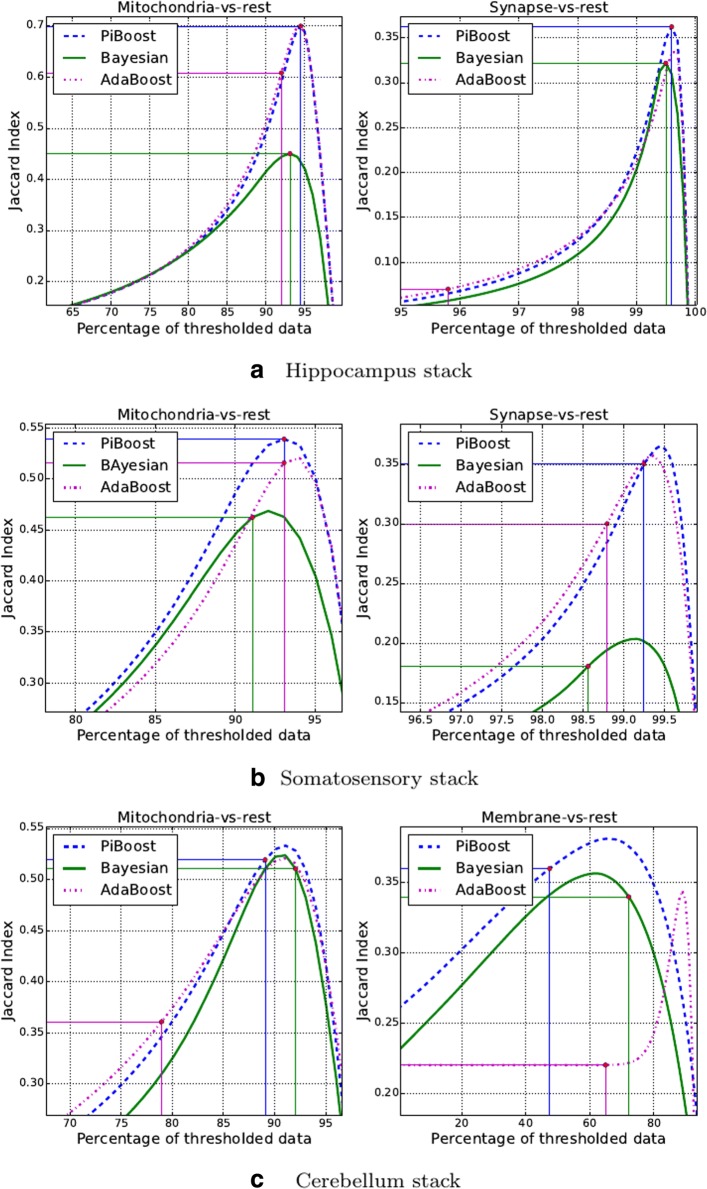
Mitochondria, synapses and membranes JCCs for the Hippocampus (**a**), Somatosensory (**b**) and Cerebellum (**c**) SBFEM image stacks. In each curve we show with a red dot the zero threshold operating point for Boosting classifiers and MAP point for the Bayesian one. JCCs let us compare the segmentation performance regardless of the operating point. The higher the curve the better the segmentation algorithm

In Fig. 8 we show the JCC curves resulting from the segmentation of mitochondria, synapses and membranes in each of the previously described image stacks. Traditionally, segmentation results have been compared by choosing an appropriate classification threshold for the classifier and computing the Jaccard for the result. This is equivalent to selecting an operation point in the JCC. This operation point for Boosting algorithms is given by a sgn() function, i.e. a zero classification threshold [31], whereas in the Bayesian classifier case it is a Maximum a Posteriori (MAP) rule [31]. In the first two columns of Table 3 we give the Jaccard index resulting from segmenting the image at this operation point. Moreover, in each curve in Fig. 8 we show with a red dot the operation point for each classifier. In some circumstances, such as for example when classes are very unbalanced, the zero threshold of Boosting algorithms may be fine-tuned [29]. This threshold is an important parameter for reproducibility and should only be estimated on a separate validation set, never in the test set. In our analysis we do not adjust it since the JCC already provides information for all thresholds.

**Fig. 9:**
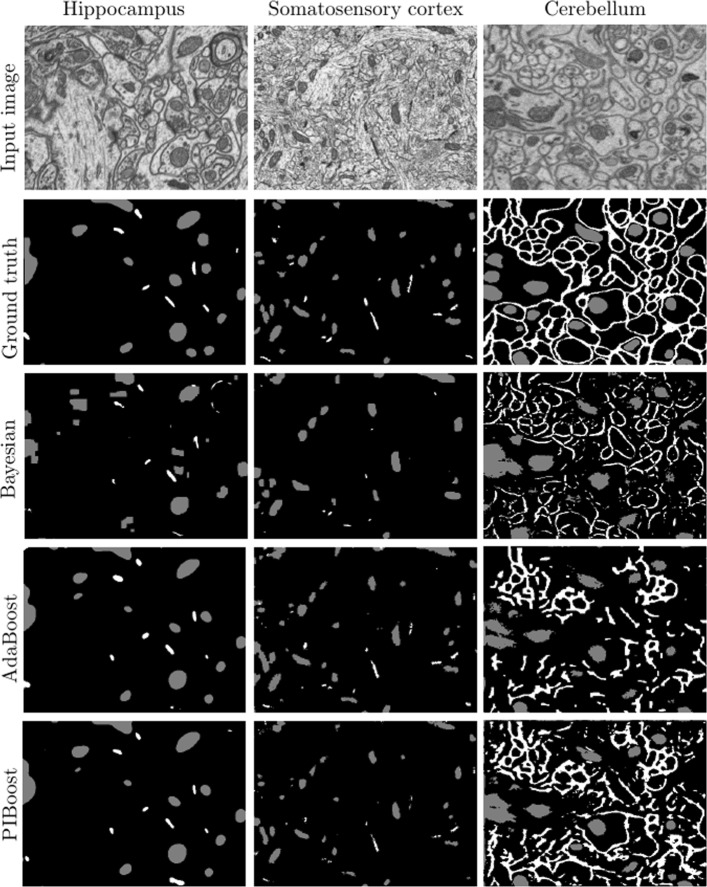
Segmentation results for each stack and algorithm after regularization. Best qualitative appreciation of segmentation differences among algorithms and image stacks by zooming in the electronic version of the paper

**Table 3:** Quantitative segmentation results for mitochondria, synapses and membrane on the Hippocampus, Somatosensory Cortex and Cerebellum stacks evaluated with the Jaccard index

	Segmentation		Two-class regularization		*αβ*-swap regularization	
	Mitochondria	Synapses	Mitochondria	Synapses	Mitochondria	Synapses
PIBoost	0.70	0.36	0.73	0.41	0.76	0.34
AdaBoost [14, 16]	0.62	0.07	0.71	0.30	-	-
Bayesian [28]	0.45	0.32	0.46	0.29	0.49	0.28
Hippocampus Stack						
	Mitochondria	Synapses	Mitochondria	Synapses	Mitochondria	Synapses
PIBoost	0.54	0.35	0.57	0.41	0.57	0.31
AdaBoost [14, 16]	0.51	0.30	0.55	0.39	-	-
Bayesian [28]	0.46	0.18	0.47	0.17	0.52	0.28
Somatosensory Cortex Stack						
	Mitochondria	Membrane	Mitochondria	Membrane	Mitochondria	Membrane
PIBoost	0.52	0.36	0.57	0.31	0.56	0.31
AdaBoost [14, 16]	0.36	0.22	0.55	0.29	-	-
Bayesian [28]	0.51	0.34	0.55	0.21	0.52	0.28
Cerebellum Stack						

From the analysis of the JCC curves in Fig. 8 we can see that, in general, the approach presented in this paper, based on the PIBoost classification algorithm and a set of pooled GRIMS features, achieves equal or superior performance to all other approaches in all stacks and structures.

In the segmentation of both mitochondria and synapses context information plays a key role. For this reason the AdaBoost and PIBoost approaches, both based on pooled channel features, achieve the best performance on both structures in the perfectly isotropic Hippocampus stack (see Fig. 8a and Table 3). The Somatosensory and Cerebellum stacks are increasingly an-isotropic. In this case most of the close context information across slices is lost and pooled channel features become less informative. Hence, segmentation performance degrades, specially for mitochondria. However, since GRIMS channels acquired at different scales also provide some local context information, the segmentation algorithm based on PIBoost degrades to a lesser extent (see Table 3).

Finally, the segmentation of membranes on the most an-isotropic Cerebellum stack (see Fig. 8c) does not depend on context but on local appearance. This is due to the fact that membranes are distributed all over the stack and have very different context. In this case, the algorithms based on GRIMS, PIBoost and Bayesian approaches, provide the best performance in the classification stage. We have also evaluated membrane segmentation results using the *Rand F-score*(${\mathcal {F}}_{r}$) and the *Information Theoretic F-score*(${\mathcal {F}}_{it}$) [35] (see Table 4). Here again the approach based on combining the simultaneous segmentation of the two possible classes, PIBoost, outperforms the rest.

**Table 4:** Quantitative segmentation results for membrane Cerebellum stack evaluated with the *Rand F-score*(${\mathcal {F}}_{r}$) and *Information Theoretic F-score*(${\mathcal {F}}_{it}$) metrics

	${\mathcal {F}}_{r}$	${\mathcal {F}}_{it}$
PIBoost	0.93	0.91
AdaBoost [14, 16]	0.81	0.84
Bayesian [28]	0.83	0.82
Cerebellum Stack		

After classifying each voxel we regularize the resulting labels with two standard graph-cut-based algorithms. This regularization usually boost the performance and visually improves the results for large and regular regions such as mitochondria (see Fig. 9). However, with thin and elongated structures like synapses and membranes, graph-cut regularization can be detrimental. This may be appreciated in the regularized results for membranes in Table 3. Some approaches use a regularization scheme that has been specifically conceived for the segmentation problem addressed. This is the case, for example, of the regularization approach used for segmenting mitochondria in [16]. In the case of multi-class classifiers like PIBoost and Bayesian, our proposed regularization scheme uses both the multi-class *αβ*-swap algorithm and, by posing it as two bi-class problems, the two-class graph-cut. The former is similar to the regularization used in [28]. For the two-class AdaBoost algorithm we use a graph-cut-based regularizer. Analyzing the segmentation results before and after regularization lets us make a fair comparison with [14], that uses no regularizer. However, as discussed above, for mythocondria segmentation, the approach in [16] uses a different type of regularizer. With this specially conceived regularization scheme [16] achieves on the Hippocampus stack a Jaccard of 0.74, slightly better than the result with our standard grap-cut-based scheme, but still behind 0.76 achieved with PIBoost using *αβ*-swap (see first row in Table 3).

In the next experiment we further analyze the reason why our algorithm achieves a good segmentation accuracy. To this end we select the Hippocampus stack, for which the AdaBoost-based approach of Lucci et al. [16] is the state-of-the-art for mithocondria segmentation. The first row in Table 5 shows the results for the AdaBoost classifier with the integral channel features on the set of channels described in [16] with a zero-threshold classification. Only by changing the channels to GRIMS and the elliptical descriptor, we get a small improvement for mitochondria and an large improvement in synapses. This is due to the fact that the elliptical descriptor and GRIMS features provide multi-scale information fundamental for the estimation of synapses, specifically those near the borders of the stack. By changing the two-class classifier for a multi-class Boosting approach we get a new improvement in performance for both structures, as shown in the third row of Table 5. The final boost in performance for our approach comes from selecting the best GRIMS scales, as shown in the last row.

**Table 5:** Hippocampus segmentation results (Jaccard) for different classifier, features and GRIMS scales

Classifier	Features	Mitochondria	Synapses
AdaBoost	EPFL [14, 16]	0.618911	0.071279
AdaBoost	GRIMS+El	0.622995	0.282041
PIBoost	GRIMS+El	0.691545	0.347415
PiBoost	GRIMS+El+	0.705145	0.364175
	Scale Selection		

The results for synapses segmentation in the Hippocampus stack in Table 5 are worse than those in [14]. Here we analyze this discrepancy. The poor result in our experiment is caused by two factors. First and foremost, the fact that the zero threshold operation point is particularly harmful for this problem (see Fig. 8a). The reason for this is that AdaBoost performs poorly on highly imbalanced classification problems, such as synapses segmentation. PIBoost, on the other hand, achieves better performance because it was conceived to address the imbalanced situations arising in multi-class classification [30]. Second, the information provided by the context cues features degrades in those voxels near the borders of the stack, since many of the cubes straddle the stack limits (Fig. 5b graphically depicts this problem). This is in part alleviated by the local information provided by the GRIMS. To evaluate the impact of these issues on the results we peel off from the Jaccard index computation in the stack the 10 voxels thick outer rind. In this case the Jaccard increases to 0.37. If we further overfit the test data and select the best operation point, the performance goes up to 0.54, comparable with that in [14].

Concerning the computational cost at run-time, the multi-class Boosting approach is computationally more efficient than the AdaBoost binary solution. However, the Bayesian approach is, by a large margin, the fastest algorithm. We have made these performance experiments on a computer with an Intel Xeon CPU at 2.40 GHz, 96 GB RAM. The PIBoost solution involves 3 separators, composed of 50 trees of depth 10. So, the classification of one voxel involves 3 separators ×50 trees in each separator ×10 inner node decisions in each tree. This is a total of 10×50×3=1500 image measurements to classify one voxel. This classifier takes 90.4 min to label the test images in the Hippocampus stack. The AdaBoost binary solution is based on two classifiers, one for each positive class, each composed of 1200 decision stumps. Hence, to classify one voxel we have to sample 1200×2=2400 image values. This means that his classifier requires 128.7 min to label the test images in the Hippocampus stack.

The Bayesian approach trains a multi-class classifier with a feature vector of 5 features ×4 scales, 5×4=20 features. It uses 2.7 min to label the test images in the Hippocampus stack.

## Conclusions

In this paper we have presented an algorithm for segmenting mitochondria, synapses and membranes in SBFEM images of brain tissue.

We have shown that the segmentation accuracy in SBFEM images can be improved by simultaneously analyzing several neuronal structures. We successfully tackled this problem using PIBoost [30], a boosting algorithm for class-imbalanced problems.

We have also verified that when the set of segmented structures have different sizes, selecting a good set of scales for image description significantly improves the segmentation accuracy. To this end we have introduced a new multi-class feature selection algorithm.

Following previous results in the literature [14, 16, 28], we have also confirmed the importance of context for segmenting neuronal structures. Although pooled channels with standard image features [14, 16] provide excellent performance in the central part of an isotropic stack, we have proved that GRIMS provide better overall performance both in isotropic and anisotropic stacks due to their capacity to represent multi-scale information.

Considering the computational cost of the classifiers, if accuracy is the main requirement in the segmentation process, then PIBoost should be the selected classifier, since it provides the best accuracy at computational cost lower than AdaBoost. However, if computational efficiency is the main issue, then the statistical approach in [28] is, by a far margin, the fastest.

The results in this paper are relevant to the neuroscience research community when confronting the reconstruction of the “synaptome” [9]. Firstly, because the methodology introduced in the paper is general and may be applied to segment different neuronal structures, possibly using other imaging modalities. Secondly, because when the number of neuronal structures to segment grows, if the segmentation problem is addressed one structure at a time, the computational requirements also grow, at least, linearly. However, using a simultaneous segmentation approach, the number of required features and, hence, the computational cost, increases at a slower pace, since many of these features may be shared by several structures. Moreover, since these features are selected to discriminate among a large group of structures they are more general and also achieve better segmentation accuracy, as we have confirmed in our experiments.

## Abbreviations

GRIMS: Gaussian Rotation Invariant MultiScale descriptors (described in the paper)
JCC: Jaccard Curve (defined in the paper)
MRF: Markov Random Field
PR: Recision-Recall curve
ROC: Receiver Operator Charateristic curve
SBFEM: Serial Block Face Electron Microscopy

## Footnotes

1 The Hippocampus stack was annotated at the École Polytechnique Fédérale de Lausanne (EPFL) under the supervision of Prof. Graham Knott. The Somatosensory stack was annotated at the Cajal Cortical Circuits laboratory, Universidad Politécnica de Madrid (UPM), under the supervision of Prof. Javier de Felipe.

**Table 2:** Data sets used in the experiments

Dataset	Labels	Voxel size (nm)	Train (voxels)	Test (voxels)
Hippocampus	Mit & Syn	5×5×5	1024×653×165	1024×653×165
Somatosensory cortex	Mit & Syn	14.7×14.7×20	366×494×100	366×494×113
Cerebellum	Mit & Mem	10×10×50	700×700×20	700×700×26

We denote the annotated labels in each data set as synapses (Syn), mitochondria (Mit) and membranes (Mem). We select train and test sets from consecutive slices in the stack. We use approximately half of each stack for training and the rest for testing

2 More information online in the Cell Centered Database (http://ccdb.ucsd.edu) with ID 8192.
